# Retrospective analysis of nontraumatic subdural hematoma incidence and outcomes in Egyptian patients with end-stage renal disease on hemodialysis

**DOI:** 10.1080/0886022X.2021.1979038

**Published:** 2021-09-22

**Authors:** Ahmed Fayed, Ayman Tarek, Mohamed I. Refaat, Sameh Abouzeid, Sohail Abdul Salim, Lajos Zsom, Tibor Fülöp, Karim M. Soliman, Mohamed A. Elmallawany

**Affiliations:** a Nephrology Unit, Internal Medicine Department, Kasr Alainy School of Medicine, Cairo University, Cairo, Egypt; b Neurosurgery Department, Kasr Alainy School of Medicine, Cairo University, Cairo, Egypt; c Nephrology Department, Theodor Bilharz Research Institute, Cairo, Egypt; d Department of Medicine, Division of Nephrology, University of Mississippi Medical Center, Jackson, MS, USA; e Fresenius Medical Care Hungary, Cegléd, Hungary; f Department of Medicine, Division of Nephrology, Medical University of South Carolina, Charleston, SC, USA; g Medical Services, Ralph H. Johnson VA Medical Center, Charleston, SC, USA; h Department of Surgery, Transplant Nephrology, Medical University of South Carolina, Charleston, SC, USA

**Keywords:** Hemodialysis, mortality, nontraumatic subdural hematoma, outcome

## Abstract

**Background:**

The incidence of subdural hematoma (SDH) in chronic maintenance hemodialysis (CMH) patients may change over time, along with the evolving characteristics of the underlying populations.

**Methods:**

We conducted a retrospective, single-center study at Cairo University hospitals, assessing the incidence, associated risk factors, and outcomes of nontraumatic SDH in CMH patients between January 2006 and January 2019.

**Results:**

Out of 1217 CMH patients, nontraumatic SDH was diagnosed in 41 (3.37%) during the study, increasing with the enrollees’ age but stable over the observation period and translating into an annual incidence rate of 28 per 1000 patients per year. SDH patients were likely to use central venous catheters, reported pruritis and history of bone fractures, and had higher phosphorus, parathyroid hormone, and alkaline phosphatase values (*p* < 0.001); however, there was no association with atrial fibrillation or use of anticoagulants. In the SDH cohort (*n* = 41), six patients did not need surgical intervention and 13 patients died before becoming surgically fit for intervention; mortality correlated with ischemic heart disease (*p* = 0.033) and the presence of atrial fibrillation or chronic anticoagulation with warfarin (*p* < 0.0001 for both), among others. Twenty-two patients received surgical operations and of these 2 died postoperatively; overall patient mortality was 12/41 (29.27%) at 30 days and 15/41 (36.59%) at 1 year.

**Conclusion:**

Our study demonstrated a striking enrichment for underlying comorbidities in those patients developing SDH and a high risk of immediate mortality. The benefit of chronic anticoagulation therapy should be carefully weighed against the risk of CNS bleed in MHD patients.

## Introduction

In dialysis patients, cerebrovascular disease is common, often diagnosed late, and carries a substantial risk of morbidity and mortality. Among these, stroke is the third most common cause of death and cardiovascular disease is the leading cause of death; however, characteristics of cerebrovascular disease – including clinical subtypes and associated effects – are not well described in the literature [1].

Subdural hemorrhage (SDH) is generally more common in elderly patients and associated with a high rate of morbidity and mortality. Sozio et al. reported an annual incidence of chronic SDH is 1–5.3 cases per 100,000 populations [1]. More recent research reports even a higher incidence, perhaps due to improved imaging techniques [1,2]. SDH is also known to be a complication of long-term hemodialysis, and prevalence in chronic maintenance hemodialysis (CMH) patients doubled in recent years, likely attributable to the increased utilization of oral anticoagulant therapy [1,2]. Sood et al. also reported a 10-fold increased risk of developing SDH in CMH patients compared with that in the general population [3]. Moreover, one study in Japan reported that in patients on CMH, SDH accounts for as much as 8.6% of sudden deaths [4].

Because of multiple studies reporting a wide variance in incidence in CMH as well as insufficient data on risk factors and outcomes, we wished to explore these characteristics of SDH in our CMH population.

## Patient and methods

This single-center, retrospective cohort study included data of all the adult subjects who were diagnosed with CMH at the Cairo University Hospital’s affiliated renal centers between 2006 and 2019. Kasr Al-Ainy University Hospital is a major hospital and tertiary referral center that serves patients from Cairo and all other governorates in Egypt. The University Hospitals’ three renal centers offer nephrological services to a large population with a total of more than 10 000 hemodialysis sessions per year.

All paper and electronic records related to admission, inpatient stay, laboratory tests, radiological investigations, and inpatient and outpatient dialysis data were analyzed. Baseline demographics and comorbidities such as heart failure, diabetes mellitus, essential hypertension, and atrial fibrillation with or without anticoagulant use were identified and confirmed during the follow-up period (Table 1). All cases of SDH were reviewed by the neurosurgical team at our center with regards to eligibility for surgical treatment; laboratory data were obtained at the initial presentation at an emergency room. In keeping with best surgical practice, guidelines for surgical evacuation in SDH were midline shift >5 mm or hematoma thickness >10 mm, regardless of the level of the Glasgow Coma Scale (GCS) [5]. During routine care, unfractionated heparin was used for all our patients on CMH and administered as a 500 IU bolus at the start of dialysis, followed by a maintenance dose of 500 IU every hour thereafter, plus an additional 50 IU for every 1 kg above ideal body weight in both patients’ bolus and maintenance doses. Dialysis session length varied from 4 to 6 h. Dialysis adequacy was measured monthly by single-pool Kt/V (spKt/V) using the Daugirdas method. Dialysis prescription was tailored to achieve an spKt/V of ≥1.6.

**Table 1.** Demographic and laboratory data of our patients received maintenance HD and patients experienced nontraumatic subdural hematoma (SDH).

Variables	Without non-traumatic SDH [1176]	With non-traumatic SDH [41]	*p*-Value
**Age (**Years**)** (Mean ± SD)	50.3 ± 13.8	56.3 ± 14.3	0.0064
**Smoking** (Number (%))	339 (28.8)	6 (14.6)	0.0474
**Female** (Number (%))	595 (50.6)	18 (44)	0.4062
**Male** (Number (%))	581 (49.4)	23 (56)	0.4031
**Major co-morbidity** (Number (%))			
1. Diabetes mellitus	767 (65.2)	28 (68.3)	0.6820
2. Systemic hypertension	937 (79.7)	32 (78.1)	0.8026
3. Ischemic Heart Disease	474 (40.3)	14 (34.2)	0.4336
4. Malignancy	56 (4.8)	0 (0)	0.1510
5. Fractures	28 (2.4)	5 (12.2)	0.0002
History of prior fractures	26 (2.2)	5 (12.2)	0.0001
fractures on arrival or within <30 days	2 (0.2)	0 (0)	0.7745
6. Atrial fibrillation	234 (19.9)	10 (24.4)	0.4795
7. Thrombosis	14 (1.2)	0 (0)	0.4807
8. Use of oral anticoagulants (warfarin)	248 (21.1)	10 (24.4)	0.6115
9. Use of aspirin/clopidogrel	937 (79.7)	32 (78.1)	0.8026
10. Hepatitis C virus	345 (28.3)	5 (12.2)	0.0237
11. Hepatitis B virus	3 (0.3)	2 (2.8)	0.0110
12. Urine volume ≤400 ml	980 (83.3)	39 (95.2)	0.0427
13. Urine volume >400 ml	196 (16.7)	2 (2.8)	0.0177
**Dialysis frequency/time:**	Three sessions weekly each session 4 h		
**Dialysis vintage, in years** (Mean ± SD)	3.4 ± 0.2	2.8 ± 2.3	<0.0001
Vascular Access (Number (%))			
1. Arterio-Venous Fistula	1049 (89.2)	26 (63.4)	<0.0001
2. Graft	77 (6.5)	1 (2.4)	0.2906
3. Tunneled Dialysis Catheters	50 (4.3)	14 (34.2)	<0.0001
Laboratory data (Mean ± SD)			
Hemoglobin conc. (12-15.5 g/dL)	9.9 ± 1.8	11 ± 2.4	0.0002
Platelet count (150–450 × 10^9^/ml)	260 ± 70.1	173 ± 75	<0.0001
Ferritin (20–250 ng/ml)	326 ± 60	162 ± 21.7	<0.0001
PT (11–13.5 seconds)	13 ± 0.9	11.4 ± 0.7	<0.0001
APTT (30–40 seconds)	32.2 ± 2.9	32.5 ± 3.2	0.5166
Fibrinogen (2–4 g/L)	4.4 ± 0.5	4.22 ± 0.74	0.0264
Serum albumin (3.4–5.4 g/L)	3.2 ± 0.4	3.3 ± 0.5	0.1192
Corrected calcium (8.6–10.2mg/dl)	8.3 ± 0.9	8.5 ± 0.7	0.1594
Phosphorous (3–4.5 mg/dl)	5 ± 0.8	6 ± 2	<0.0001
Alkaline phosphatase (44–147U/L)	137.4 ± 7.5	156.9 ± 79.7	<0.0001
Serum parathormone (10–55 pg/mL)	355.5 ± 74.2	420.2 ± 93.1	<0.0001
Uric Acid (2.4–6 mg/dl)	5.4 ± 1.6	6.8 ± 2.7	<0.0001
Single pool Kt/V (1.2)	1.5 ± 0.4	1.4 ± 0.3	0.1132

PT: prothrombin time; APTT; activated partial thromboplastin time. * *p* < 0.05 are significantly different.

All processes carried out in this study involving human subjects followed the ethical principles of the institutional and/or national research committee, the Declaration of Helsinki of 1964, and the latter’s corresponding amendments or similar ethical criteria. All individual participants participating in the study received informed consent. The local ethical committee of the Department of Internal Medicine, School of Medicine, Cairo University, approved this work in December 2019.

### Statistical analysis

Precoded data were entered on the computer using Microsoft Excel 2010 for Windows. Data were then transferred to the Statistical Package of Social Science Software program (SPSS-25) to be statistically analyzed. Continuous variables were expressed as mean ± standard deviation. Frequency and percentage were done for qualitative variables. Comparisons between quantitative variables were done using the nonparametric Kruskal–Wallis and Mann–Whitney tests [6]. For comparing categorical data, a chi-squared (*χ*^2^) test was performed. When the expected frequency was less than 5, Fisher’s exact test was used instead [7]. Correlations between quantitative variables were done using the Spearman correlation coefficient [8]. A *p*-value of less than 0.05 was considered statistically significant.

## Results

**Figure 1.**
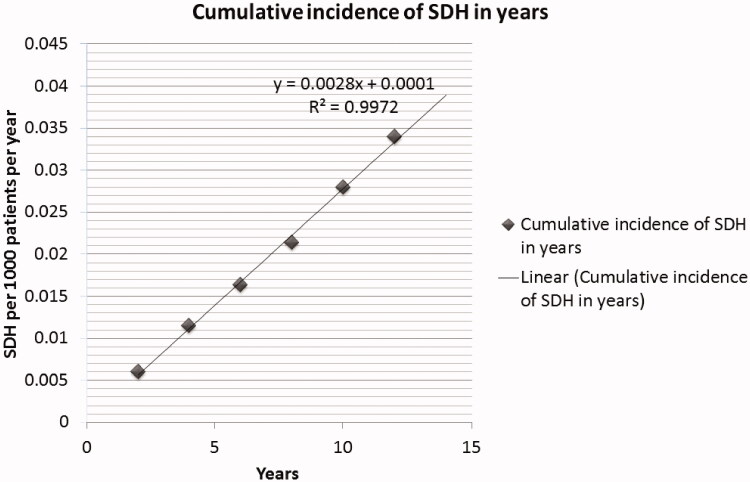
Cumulative incidence of SDH in years.

Demographic and laboratory data of our patients are shown in Tables 1 and 2. Systemic hypertension was the most common cause of ESRD as well as the major co-morbidity. Non-traumatic SDH in CMH patients was found to be 0.4% prevalent at our center, with an annual incidence of 189 per 100,000 patients, compared to the prevalence previously recorded in nonhemodialysis patients (1–5.3 cases per 100,000 populations). Out of 1217 CMH patients, nontraumatic SDH developed in 41 (3.37%) during the study period, translating to an annual incidence rate of 28 per 1000 patients per year. SDH incidence was associated with increased age of the patient (the age of CMH patients was 50.7 ± 14.2 whereas the age of nontraumatic SDH was 50.3 ± 13.8, *p*-value 0.0064). We found a good linear relationship between the cumulative incidence of SDH and time (year) (Figure 1).

**Table 2.** Comparison of survivors and non survivors in patients experienced nontraumatic subdural hematoma (SDH).

Variables	Survivors in nontraumatic SDH (Mean ± SD)	Nonsurvivors in nontraumatic SDH (Mean ± SD)	*p*-Value
Age (Years) (Mean ± SD)	55.7 ± 1.9	71.5 ± 1.4	<0.0001
Smoking (Number (%))	3 (28.4)	3 (14.6)	0.4728
Female (Number (%))	11 (50.4)	7 (44)	0.0699
Male (Number (%))	15 (49.6)	8 (56)	0.7928
Major co-morbidity (Number (%))			
1. Diabetes mellitus	18 (69.2)	10 (66.7)	0.8692
2. Systemic hypertension	19 (73.1)	13 (86.7)	0.3234
3. Ischemic Heart Disease	12 (46.2)	2 (13.3)	0.0332
4. History of prior fractures	5 (19.2)	0 (0)	0.0729
5. Atrial fibrillation	1 (3.8)	9 (60)	0.0001
6. Use of oral anticoagulants (warfarin)	1 (3.8)	9 (60)	0.0001
7. Use of aspirin/clopidogrel	22 (84.6)	10 (66.7)	0.19001
8. Hepatitis C virus	4 (15.4)	1 (6.7)	0.4239
9. Hepatitis B virus	2 (7.7)	0 (0)	0.29909
10. Urine volume ≤400 ml	24 (92.3)	15 (100)	02822
11. Urine volume >400 ml	2 (7.7)	0 (0)	0.29909
Subdural thickness (Number (%))			
Subdural thickness >20 mm	15 (57.7)	13 (86.7)	0.0576
Subdural thickness >10 mm associated midline shift	11(42.3)	2 (13.3)	0.0576
Laboratory data (Mean ± SD)			
Hemoglobin conc. (12–15.5 g/dL)	12.1 ± 1.3	9.5 ± 0.7	<.00001
Platelet count (150–450 × 10^9^/ml)	181.3 ± 9.2	144 ± 6.8	<.00001
Ferritin (20–250 ng/ml)	201.3 ± 35.8	314.5 ± 14.1	<0.0001
PT (11–13.5 seconds)	11.5 ± 0.3	11.6 ± 0.1	0.01701
APTT (30–40 seconds)	32.3 ± 1.7	32.8 ± 0.1	0.30276
Fibrinogen (2–4 g/L)	4.3 ± 0.1	4.4 ± 0.1	0.00076
Serum albumin (3.4–5.4 g/L)	3.5 ± 0.2	2.9 ± 0.1	<0.0001
Corrected calcium (8.6–10.2mg/dl)	8.6 ± 0.2	8.5 ± 0.2	0.7537
Phosphorous (3–4.5 mg/dl)	6.1 ± 0.5	6.4 ± 0.3	0.01812
Alkaline phosphatase (44–147U/L)	162.3 ± 5.9	163.2 ± 4.7	0.6049
Serum parathormone (10–55 pg/mL)	427.1 ± 21.6	419.3 ± 18.2	0.2475
Uric Acid (2.4–6 mg/dl)	6.9 ± 0.3	6.9 ± 0.3	0.9057
Single pool Kt/V (1.2)	1.3 ± 0.1	1.3 ± 0.1	0.6952

PT: prothrombin time; APTT; activated partial thromboplastin time. *p < 0.05 are significantly different.

Occurrence of nontraumatic SDH in CMH patients was statistically more likely with shorter dialysis vintage (*p* < 0.001), use of central venous catheters (*p* < 0.001), and reported history of past bone fractures (*p* = 0.0001) but not with atrial fibrillation or use of anticoagulants (Table 1). Moreover, those with SDH had significantly lower hemoglobin and lower ferritin values (*p* < 0.001) but higher phosphorus, parathyroid hormone, and alkaline phosphatase (Table 1). Among those with SDH, death was more likely observed in those with underlying ischemic heart disease (*p* = 0.033) and the presence of atrial fibrillation and chronic anticoagulation (*p* < 0.001) (Table 2).

Of the 41 patients, six patients had a small hematoma that was not large enough for surgical intervention; 13 patients died before being surgically fit for intervention; and 22 patients received surgery, of these 2 died postoperatively (Table 3). SDH thickness >20 mm was found in 28/41 (68.29%) cases, whereas 13/41 (31.7%) cases had a significant (>10 mm) associated midline shift. Patient mortality was 12/41 (29.27%) at 30 days and 15/41 (36.59%) at 1 year. At 1 year, 13/15 (70.73%) of patient deaths occurred in patients deemed not eligible for surgical intervention with an SDH thickness >20 mm and/or midline shift >10 mm. All these patients died as a direct result of SDH. Of the 22 cases who underwent surgery, 15 (68%) had acute SDH, four (18%) had subacute SDH, and three (14%) had chronic SDH. The subacute and chronic cases (7 patients) underwent burr hole evacuation whereas the acute cases (15 patients) underwent a decompressive flap and duroplasty. Among these, two died and 13 survived (Table 3).

**Table 3.** Indications, interventions, surgical techniques and outcomes of survivors and nonsurvivors in patients experienced nontraumatic subdural hematoma (SDH).

Variables	Survivors in nontraumatic SDH (Number (%))	Nonsurvivors in nontraumatic SDH (Number (%))	*p*-Value
Surgical intervention was not done in 19 cases (46.3%)			
The hematoma was not sizable enough	6 (23.1)	0 (0)	0.0466
Not fit for surgical intervention	0 (0)	13 (86.7)	<0.0001
Surgical intervention was done in 22 cases (53.4%)			
Acute subdural hematoma	13 (50)	2 (13.3)	0.0203
Sub-acute and chronic SDH	7 (26.9)	0 (0)	0.0294
The technique of surgical intervention			
Burr-hole evacuation	7 (26.9)	0 (0)	0.0294
Decompressive flap and duroplasty	13 (50)	2 (13.3)	0.0203
**Total mortality rate**			
At 30 days	12 (80)		0.0012
At 1 year	3 (20)		

*p < 0.05 are significantly different.

## Discussion

This retrospective observational study reaffirmed the high incidence and mortality of SDH in CMH patients, particularly in the elderly. Moreover, our study also demonstrated a striking enrichment of underlying comorbidities in those patients who developed SDH. Overall mortality results in our population are comparable to studies on CMH patients from the United States reporting 29.27% and 4.88% mortality rates at 30 days and after 30 days, respectively [9]. In concordance with our results, Wang et al. reported a 35.2% 30-day mortality rate in CMH patients who develop SDH [10].

The higher incidence of SDH in CMH patients can be explained by several factors. The risk of bleeding is increased by the use of heparin with dialysis, as well as by concurrent administration of warfarin or antiplatelet for cerebrovascular diseases, cardiovascular diseases, or atrial fibrillation. Increased risk of bleeding can also be attributed to the uremic state itself. The etiology for increased bleeding tendency in the uremia is not completely understood but impaired platelet function is one of the main determinants of uremic bleeding [11]. This impairment is largely due to incompletely defined inhibitors of platelet function in the plasma of patients with markedly reduced kidney function. Abnormal platelet–endothelial interaction and anemia also play a role [5, 12].

Further, Rossier et al. found that elderly dialysis patients are at higher risk compared with the general population or compared with young CMH patients [13]. Possibly explanations for this propensity may include protein malnutrition, vitamin D deficiency, mineral bone diseases, cognitive impairment, and post-dialysis hypotension, which may all increase susceptibility for falls in CMH patients [14]. In patients with ESRD, brain atrophy is common, affects as much as 77.5% of those with CMH, and will increase the length of the bridging veins that are prone to tearing [15]. Hemodialysis has been associated with higher fluctuations in intracranial pressure, alteration in cerebral blood flow, and decreased pressure in subdural space during the intra-dialytic period [16]. Cortical atrophy may be linked to repeated hypotensive episodes in CMH patients [17,18].

In concordance with other studies, we demonstrated an increased risk of death in nontraumatic SDH in CMH patients with ischemic heart disease, atrial fibrillation, and the use of oral anticoagulants (*p*-value < 0.001) [3,19]. The presence of an anticoagulated state will increase the length of time needed for reversal of anticoagulant effect during preoperative preparation and allows the increase in the size of hematoma during this time interval. It is also possible that the presence of oral vitamin-K antagonist may contribute to tissue fragility beyond the simple anticoagulation effect, *via* interfering with matrix Gla protein synthesis [20].

Factors that have been found to increase the vulnerability to falls for CMH patients include advanced age, comorbidities, cognitive impairment, psychotropic drug use, blood pressure drug use, malnutrition, post-dialysis hypotension, renal bone diseases, and vitamin D deficiency. Cognitive dysfunction can result from uremia itself and neuropsychiatric adverse effects from widely used drugs are likely to occur in patients with end-stage renal disease (ESRD), including those on dialysis [17]. Falls even without fractures have also been related to an increased risk of traumatic SDH in CMH patients [2]. These findings were aligned with our results, which demonstrated the statistically higher incidence of non-traumatic SDH in CMH patients with diabetes mellitus, systemic hypertension, ischemic heart disease, atrial fibrillation, the use of oral anticoagulants, the presence of fractures, and dysfunction in bone minerals such as low serum albumin, low serum calcium, high alkaline phosphatase, high serum parathormone, elevated uric acid, and high spKt/V. We found a statistically higher incidence of nontraumatic SDH in CMH patients with hepatitis C virus and hepatitis B virus infections. This seemed to be linked to the fact that these subjects are more vulnerable to more severe arterial calcification [21].

Subacute and chronic subdural hematomas were handled by wide burr holes fashioned to reach the entire extensions of the hematoma due to its liquefied blood content due to enzymatic fibrinolysis.

The burr hole should be wide enough (more than 2.5 cm in diameter) to allow continuous free drainage and prevent recurrence with careful dura coagulation to shrink its edges back to full width [22]. All of the above considerations help in successful drainage and the prevention of recurrence [22]. More commonly, acute subdural hematomas are associated with more impact damage and a rapid increase in intracranial pressure, leading to efficient drainage and preventing recurrence [22]. Attention is required for the increased risk (up to sevenfold in males and 26-fold in females) of acute subdural hematoma in patients receiving anticoagulants [23]. Timing is crucial, as previous studies have illustrated the ‘four-hour rules’ in which mortality rate is 30% for posttraumatic acute SDH in cases operated within four hours compared with 90% if surgery was postponed [24]. This explains that preoperative cases died (13 cases) largely from coagulopathy because they were not prepared for surgical intervention. In conjunction with duroplasty, a decompressive craniectomy flap was performed in our cohort with at least 12 cm in diameter. Both requirements are mandatory for achieving a decrease in intracranial pressure below 20 mmHg postoperatively. Kurland et al. demonstrated a survival outcome of 86.7% in SDH cases who underwent decompressive flap and duroplasty [25]. The authors concluded that decompressive craniectomy is an efficient lifesaving way to regulate elevated intracranial pressure, which accounts for the drastic increase in the use of this procedure.

The main strength of our study is its focus on CMH patients from a contemporary cohort who developed SDH, reflecting the current co-morbid burden of MHD patients and neurosurgical practice both. This study increases the understanding of outcomes in CMH patients developing SDH and highlights the necessity of increasing efforts to conduct prospective randomized controlled trials in the field. Our study is also not without limitations. Observational studies are more prone to bias, and a retrospective nature has limited our ability to capture baseline information, including lifestyle, smoking habits, and body mass index of patients. The definitive cause of SDH could not be confirmed, possibly because of reporting bias.

## Conclusion

Our study demonstrated a striking enrichment for underlying comorbidities in those patients developing SDH and a high risk of immediate mortality. The benefit of chronic anticoagulation therapy should be carefully weighed against the risk of CNS bleed in MHD patients.
